# Translational framework for implementation evaluation and research: Protocol for a qualitative systematic review of studies informed by Normalization Process Theory (NPT)

**DOI:** 10.3310/nihropenres.13269.1

**Published:** 2022-06-13

**Authors:** Carl R May, Bianca Albers, Laura Desveaux, Tracy L Finch, Anthony Gilbert, Alyson Hillis, Melissa Girling, Roman Kislov, Anne MacFarlane, Frances S Mair, Christine M May, Elizabeth Murray, Sebastian Potthoff, Tim Rapley

**Affiliations:** 1 Department of Health Services Research and Policy, London School of Hygiene & Tropical Medicine, London, UK; 2 NIHR ARC North Thames, London, UK; 3 Institute for Implementation Science in Healthcare, Zurich, Switzerland; 4 Trillium Health Partners, Toronto, Canada; 5 Department of Nursing, Midwifery & Health, Northumbria University, Newcastle upon Tyne, UK; 6 NIHR ARC North East-North Cumbria, Newcastle upon Tyne, UK; 7 Royal National Orthopaedic Hospital, London, UK; 8 Business School, Manchester Metropolitan University, Manchester, UK; 9 NIHR ARC Greater Manchester, Manchester, UK; 10 School of Medicine and Health Research Institute, University of Limerick, Limerick, Ireland; 11 Institute of Health and Wellbeing, Glasgow University, Glasgow, UK; 12 Independent Researcher, Southampton, UK; 13 Research Department of Primary Care and Population Health, University College London, London, UK; 14 Department of Social Work, Education and Community Wellbeing, Northumbria University, Newcastle upon Tyne, UK

**Keywords:** Normalization Process Theory, Complex interventions, Implementation research, Process evaluation, Qualitative evidence synthesis

## Abstract

**Background:**

Normalization Process Theory (NPT) identifies mechanisms that have been demonstrated to play an important role in implementation processes. It is now widely used to inform feasibility, process evaluation, and implementation studies in healthcare and other areas of work. This qualitative synthesis of NPT studies aims to better understand how NPT explains observed and reported implementation processes, and to explore the ways in which its constructs explain the implementability, enacting and sustainment of complex healthcare interventions.

**Methods:**

We will systematically search Scopus, PubMed and Web of Science databases and use the Google Scholar search engine for citations of key papers in which NPT was developed. This will identify English language peer-reviewed articles in scientific journals reporting (a) primary qualitative or mixed methods studies; or, (b) qualitative or mixed methods evidence syntheses in which NPT was the primary analytic framework. Studies may be conducted in any healthcare setting, published between June 2006 and 31 December 2021. We will perform a qualitative synthesis of included studies using two parallel methods: (i) directed content analysis based on an already developed coding manual; and (ii) unsupervised textual analysis using Leximancer® topic modelling software.

**Other:**

We will disseminate results of the review using peer reviewed publications, conference and seminar presentations, and social media (Facebook and Twitter) channels. The primary source of funding is the National Institute for Health Research ARC North Thames. No human subjects or personal data are involved and no ethical issues are anticipated.

## Introduction

Implementation research looks for answers to some of the most difficult problems that we face: how to get new and improved evidence-based ways of delivering and organising healthcare into practice, and how to keep them there ^1, 2^. At the same time, implementation researchers have developed models, frameworks and theories that help us to understand, organise and evaluate the processes of implementing evidence-based innovations. Theories, in particular, offer us tools that can be used to shape understanding — and perform evaluation — of the operationalization of new technologies, techniques, and professional or organizational interventions. Normalization Process Theory (NPT) ^3– 11^ offers a set of conceptual tools that researchers and practitioners can use to analyse and understand goal-oriented action and processes in their contexts, and to inform the design of studies and analyses of these processes ^9^. 

**Figure 1.**
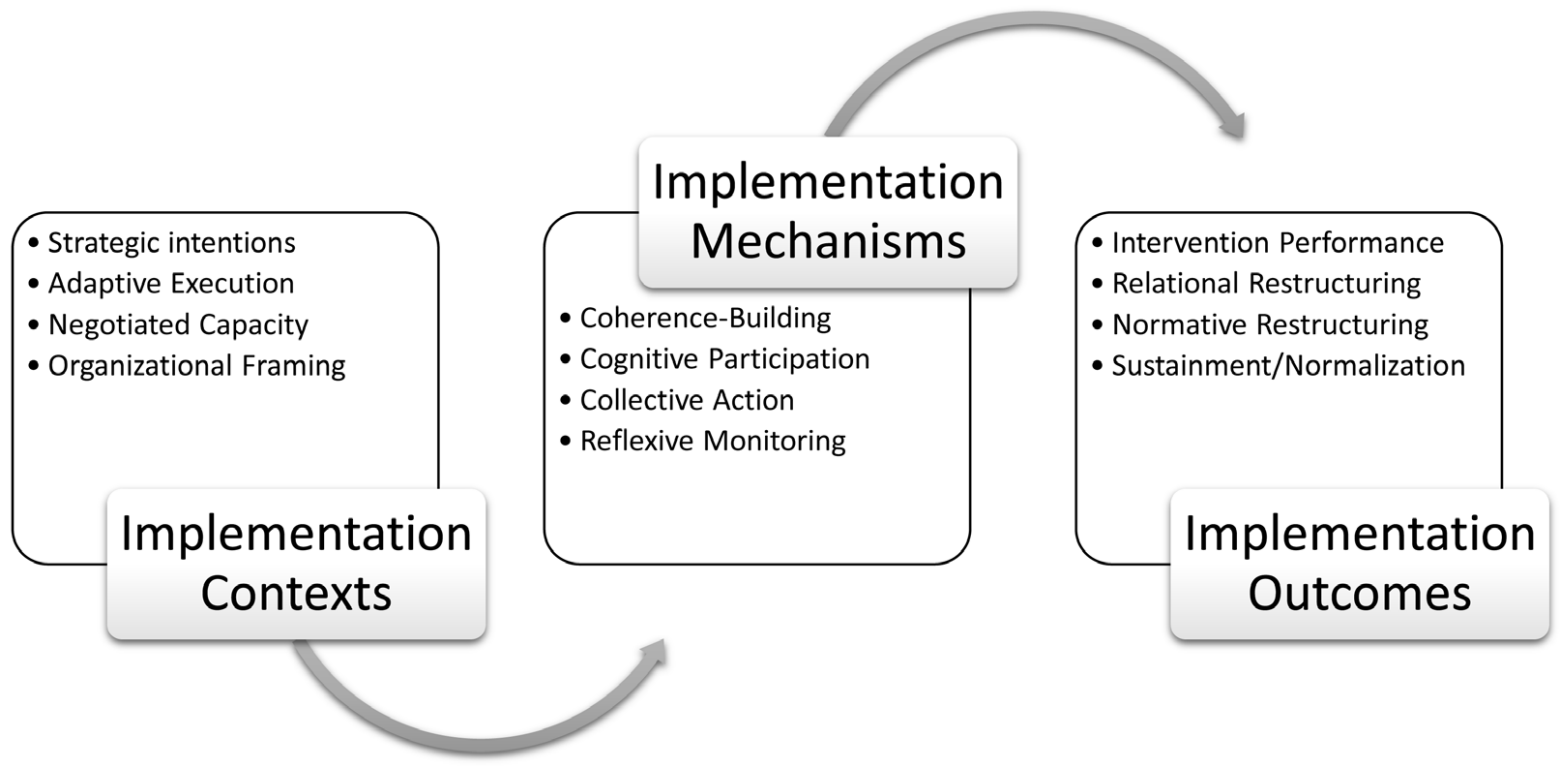
Normalization Process Theory (NPT) constructs and implementation processes.

 NPT defines implementation as the translation of strategic intentions into everyday practices through collective action and collaborative work. It identifies, characterises, and explains key features of contexts that contribute to this; the mechanisms that motivate and shape implementation processes; and key features of their outcome. The theory thus focuses on interdependent and purposive social action and identifies mechanisms that have been empirically demonstrated to play a significant role in social processes of implementation. Methodological work to support this review ^12^ has refined and consolidated the iterations of NPT developed between 2006 and 2020 ^3– 11^, into a set of 12 primary constructs. Relations between NPT constructs characterising implementation contexts, mechanisms, and outcomes are described in Figure 1. These form the basis of a generalisable coding manual for qualitative research using NPT ^12^.

This protocol sets out the foundation and procedures of a qualitative synthesis of studies that have used NPT in implementation research and process evaluation in healthcare. In an earlier review ^13^ that identified and characterised the uses and limits of NPT in research on the implementation and integration of healthcare interventions, we explored how researchers had used NPT and outlined their critiques of the theory. Interest in understanding ‘mechanisms of change’ in implementation is growing ^14– 16^, and at the same time, the value of continuous theory development has been recognised ^17– 19^. This makes it relevant to both narrow and deepen the scope of the previous review through a focused systematic review on the mechanisms of NPT, for there is much that we still do not know about implementation processes and about how they are worked out in practice.

### Aim and objectives

This qualitative synthesis of NPT studies aims to better understand how NPT explains observed and reported implementation processes, and to explore the ways in which its constructs explain the implementability, enacting, and sustainment of complex healthcare interventions. In this way, the review aims to make further progressive steps in theory development. The synthesis will answer five key research questions (RQ).

**RQ1:** How are ensembles of intervention components operationalised and enacted by their users, and how are these reported to shape their implementability?
**RQ2:** What mechanisms do NPT-informed studies identify, and how are they reported to motivate and shape implementation processes?
**RQ3:** What contextual factors are reported in NPT-informed studies, and how do they shape implementation practice?
**RQ4:** What types of outcomes are described in NPT-informed studies, and do their authors identify specific elements of the theory that cast light on this?
**RQ5**: Does qualitative content analysis suggest mechanistic differences in implementation processes between different kinds of intervention, and does it suggest that mechanisms characterised in NPT should be weighted differently or discarded in future work?

## Methods

**Figure 2.**
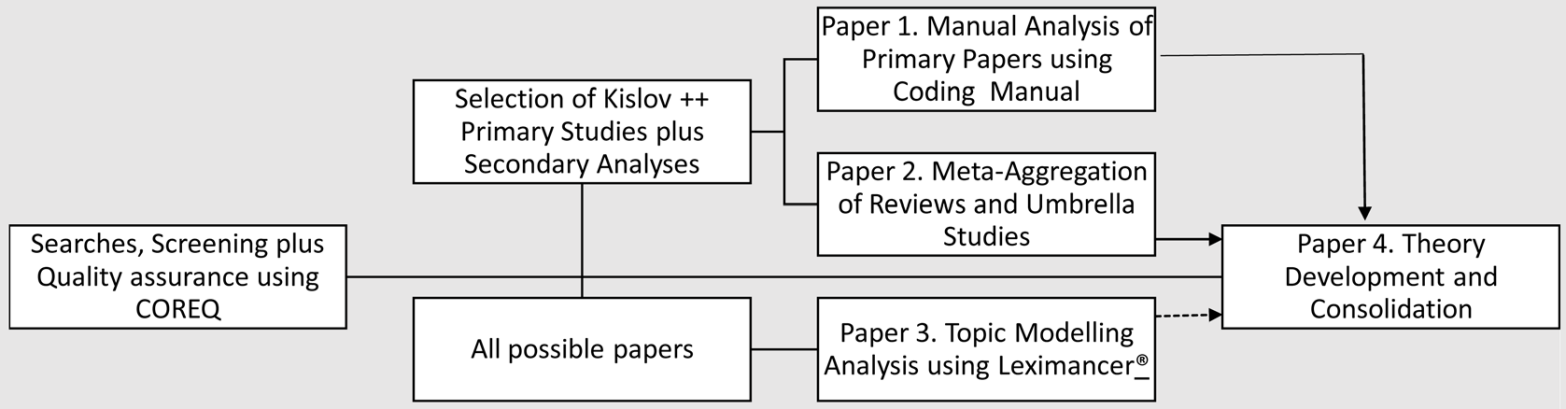
Workflow and synthesis outcome.

The design and conduct of this systematic review combines abductive ^20^ ‘manual’ qualitative analyses of primary studies that have passed quality assurance screening and that uses a coding manual based on NPT ^12^ (and which will also allow the incorporation of insights from other theories where these are used in combination with NPT); a manual mega-aggregation analysis ^21^ of reviews and other secondary studies using the same coding manual; and an automated topic modelling analysis of the whole corpus of papers using Leximancer® software (Leximancer ® Pty Ltd. Leximancer ® Qualitative Analysis Software, Release 4.5: 2009). The workflow for the programme of research is described in Figure 2.

### Registration

 Because this study reviews the development and application of an implementation theory it is not eligible for inclusion in the PROSPERO register of systematic reviews.

### Searches and citation analysis

 Our searches will update those of our earlier review ^13^. We will search three bibliographic databases (Scopus, PubMed, and Web of Science), and a search engine (Google Scholar) to find citations of papers that developed or expounded the main constructs of NPT ^3– 8, 22^; papers that developed NPT related methods or tools ^9– 11^; and citations of the NPT web-enabled online toolkit ^23^. The same search string will be used across all searches: “Normali* Process Model” OR “Normali* Process Theory” OR “general theory of implement*” OR "general model of implement*" OR “NPT Toolkit” OR “www.normalizationprocess.org”. Preliminary searches were completed from 13 ^th^ December to 22 ^nd^ December 2021 and the research team are in the process of finalising the included studies.

### Screening

In our earlier review ^13^, we discovered that combining searches using bibliographic databases (e.g., Web of Science, PubMed, and Scopus) and search engines (i.e., Google Scholar) generated not only a significant number of duplicate citations, but also significant numbers of broken or ‘page not found’ hyperlinks. Because of this, deduplication and the disposal of broken hyperlinks will be combined. One reason for this is that ‘normalization’ is also a term commonly used in a number of disciplines and is also used to describe a procedure used in the construction and reconciliation of relational databases.

Covidence © 2022 ( https://app.covidence.org), a systematic review automation tool, will be used for the screening process. AH and CRM simultaneously conducted the title and abstract screening on Covidence. Of the authors, AH has the least knowledge regarding the historical development of NPT, which will minimise bias and balance CRM’s expertise of NPT. Any studies which are eligible (i.e., they meet the criteria set out below) or which may be eligible (i.e., where the content is unclear or reviewers disagree) will be obtained in full text. Disagreements about inclusion will be resolved by discussion. The same process will be conducted for full text screening. Papers selected for inclusion will be stored as portable document format (.pdf) files in secure Endnote Libraries with automatic back up. We will characterise papers included in the review using the typology developed for our earlier review ^13^. This divided included studies into seven domains: service organisation and delivery; diagnostic and therapeutic interventions; e-Health and telemedicine; screening and surveillance tools; decision support and shared decision-making; change in professional roles; and guideline implementation. The screening results will be presented in a PRISMA flow diagram.

### Inclusion and exclusion criteria

 We will include English language peer-reviewed health and healthcare-related journal articles published between 1st June 2006 and 31st December 2021 that employ NPT either solely or in combination with some other theory to report on (a) primary studies using qualitative or mixed methods, (b) qualitative evidence syntheses (including for example qualitative systematic and scoping reviews; meta-ethnographies; and realist and hermeneutic reviews). We will exclude editorials or commentaries; protocols and other study designs; research monographs, theses or dissertations; books and book chapters; conference proceedings and abstracts; or webpages, blogs, or other social media. We will also exclude peer-reviewed studies that solely report on quantitative study designs; that contain only nominal or passing references to NPT; that are restricted to methodological or theoretical discussions or make theoretical or methodological recommendations; and reports of the application of NPT in settings other than those related to health, healthcare, and social care.

### Quality appraisal

All papers that meet the inclusion criteria outlined above will be included in analysis using Leximancer® topic modelling software by CRM. However, these inclusion criteria will generate too many papers for manual analysis. To identify papers for the latter we include papers that score ‘high’ when their quality, bias, and confidence are assessed using the Critical Appraisal Skills Programme (CASP) checklist ^24^, and that also meet the definitions developed by Kislov *et al.,* ^17, 18^ of ‘theoretically informed’ (i.e., papers that offer a rigorous non-descriptive analysis), and ‘theoretically informative’ (i.e., papers that develop relationships between theoretical constructs or challenge theoretical propositions). This will be conducted by AH with support from CRM.

### Data extraction

For all included publications, descriptive information will be extracted by AH with support from CRM, including authors, year of publication, health care problem addressed, study type and methods, data collection procedures, how NPT was used in the study, and whether this had been pre-specified in the study protocol. The extraction instrument is provided at Table 1. Procedures for the extraction of data for analysis are described below.

**Table 1.** Extraction instrument.

Category	First author and citation	Year of Publication	Country of origin	Theory frame	Domain	Research problem	Evidence base cited to support intervention	Study type Primary or Evidence Synthesis	Quality Assessment Score	Kislov *et al.* Category	Controlled / Uncontrolled Study	Application of NPT to data
Explanation				Solely NPM or NPT Combined NPM/NPT and another model, framework, or theory (e.g. CFIR) Combined NPM/NPT and methodological strategy (e.g. Participatory)	Self-care Primary Care Hospital Care Social Care (Residential) Social Care (Community) Combined/ Multiple	Professional roles and task- sets (new and changed) Patient roles and task-sets (new and changed) Professional- Patient Interactions (including telemedicine/ telecare, decision- support and shared decision- making) Treatment modalities (including diagnostic and therapeutic interventions) Informatics (e.g. Electronic Health Records and other E- Health) Screening and surveillance Guideline implementation Service organisation and delivery Other	None Primary study Systematic Review Guideline	Primary: Comparative, Longitudinal, X Sectional Secondary: Review, Meta- synthesis, Umbrella/ Meta- aggregation	High/ Medium/ Low	Theoretically informative (NPT is deployed to design an intervention and/ or to systematise, analyse or explain empirical findings, and is critiqued, refined or extended as a result. Or, if several theories are deployed, their integration, aided by empirical findings, produces novel insights that go beyond the premises of each of these theories) Theoretically informed (NPT is deployed to design an intervention and/ or to systematise, analyse or explain empirical findings. But NPT is not critiqued, refined or extended as a result) Descriptive (NPT is used to describe, or thematise/ categorise, the data. But discussion does not extend beyond descriptive themes or categories) Tokenistic (NPT is claimed as an analytic framework but the paper bears no evidence of it in use)	Yes/ No	Prospective (Theory interpretation designed into study prior to data collection) Retrospective (Interpretation in the light of Theory after data is collected)

### Data preparation

This review will combine two approaches to data analysis. We will use: (a) conventional ‘manual’ qualitative content analysis ^25^ — sometimes called ‘directed content analysis’ ^26^ — informed by the approach recommended by MacFarlane and O’Reilly de Bruin ^27^; and (b) conduct partially automated semantic and relational text searches using Leximancer® text analytic software.

Data preparation for qualitative content analysis is uncontentious. Portable document format (.pdf) copies of all included papers will be uploaded into a single NVivo® project directory (QSR International (1999) NVivo® Qualitative Data Analysis Software, release 12.0). No special data preparation is necessary for qualitative content analysis. All content analytic operations, (e.g., case identification, coding, annotation, and memoing), can be carried out using .pdf files. The results, discussions, and conclusions of included papers will be treated as formal data for the review.

Data preparation for qualitative content analysis using text analytic software modelling is not uncontentious. This is because it calls for the modification of the texts that are treated as data ^28^. This is because .pdf files present problems when they are interrogated by Leximancer®. Leximancer® software cannot easily distinguish between different forms of data and metadata, for example tables, diagrams and formatting instructions. To convert the content of .pdf files into usable data for textual analysis, text from the results, discussion, and conclusion sections of included papers will be extracted from .pdf files and converted into a separate set of Microsoft Word (.docx) files using Microsoft 365 version 2203. All metadata must be identified and eliminated from these files and text contained in tables must be extracted and reassembled as discrete paragraphs to enable Leximancer® to ‘read’ them properly ^29^.

### Qualitative data analysis

Qualitative content analysis using manual coding. Formal data for analysis will be the results, discussion and conclusion sections of included papers. This will combine abductive analysis (searching for unexpected phenomena of interest in the data), and deductive analysis using an NPT coding manual developed for this review ^12^. A synopsis of the coding manual is given in Table 2. It will be integrated into NVivo 12® Software. CRM and AH will lead the coding exercise, and all co-authors will each independently read, and check coding of included papers. Where disagreements about coding occur, they will be arbitrated by a third member of the team. 

**Table 2.** Normalization Process Theory (NPT) coding manual part A: Contexts, mechanisms, and outcomes ^12^.

Domain	NPT Construct
Implementation **Contexts** Contexts are patterns of social relations and structures that unfold over time and across settings. They make up the implementation environment.	**Strategic Intentions:** How do contexts shape the formulation and planning of interventions and their components?
	**Adaptive Execution:** How do contexts affect the ways in which users can find and enact workarounds that make an intervention and its components a workable proposition in practice?
	**Negotiating Capacity:** How do contexts affect the extent that an intervention and its components can fit, or be integrated, into existing ways of working by their users?
	**Reframing organizational logics:** How do existing social structural and social cognitive resources shape the implementation environment?
Implementation **Mechanisms** Mechanisms are revealed through purposive social action— **collective action** and **collaborative work**—that involves the investment of personal and group resources to achieve goals	**Coherence Building:** How do people work together in everyday settings to understand and plan the activities that need to be accomplished to put an intervention and its components into practice?
	**Cognitive Participation:** How do people work together to create networks of participation and communities of practice around interventions and their components?
	**Collective Action:** How do people work together to enact interventions and their components?
	**Reflexive Monitoring:** How do people work together to appraise interventions and their components?
Implementation **Outcomes** The practical effects of implementation mechanisms at work	**Intervention Performance:** What practices have changed as the result of interventions and their components being operationalized, enacted, reproduced, over time and across settings?
	**Relational Restructuring:** How have working with interventions and their components changed the ways people are organized and relate to each other?
	**Normative Restructuring:** How have working with interventions and their components changed the norms, rules and resources that govern action?
	**Sustainment (normalization):** How have interventions and their components become incorporated in practice?

**Table 3.** NPT coding manual part B: Granular codes for implementation mechanisms ^12^.

NPT construct	Sub-construct
**Coherence**: How do people work together to understand and plan the activities that need to be accomplished to put an intervention and its components into practice?	**Differentiation**: How do people distinguish interventions and their components from their current ways of working?
	**Communal specification**: How do people collectively agree about the purpose of interventions and their components?
	**Individual specification**: How do people individually understand what interventions and their components require of them?
	**Internalization**: How do people construct potential value of interventions and their components for their work?
**Cognitive Participation**: How do people work together to create networks of participation and communities of practice around interventions and their components?	**Initiation**: How do key individuals drive interventions and their components forward?
	**Enrolment**: How do people join in with interventions and their components? [48].
	**Legitimation**: How do people agree that interventions and their components are the right thing to do and should be part of their work?
	**Activation**: How do people continue to support interventions and their components?
**Collective Action**: How do people work together to enact interventions and their components?	**Interactional Workability**: How do people do the work required by interventions and their components?
	**Relational Integration**: How does using interventions and their components affect the confidence that people have in each other?
	**Skill-set Workability**: How is the work of interventions and their components appropriately allocated to people?
	**Contextual Integration**: How is the work of interventions and their components supported by host organizations?
**Reflexive Monitoring**: How do people work together to appraise interventions and their components?	**Systematization**: How do people access information about the effects of interventions and their components?
	**Communal appraisal**: How do people collectively assess interventions and their components as worthwhile?
	**Individual appraisal**: How do people individually assess interventions and their components as worthwhile?
	**Reconfiguration**: How do people modify their work in response to their appraisal of interventions and their components?

Qualitative content analysis using Leximancer® text analytic software. Leximancer® is topic modelling or text analytic software that partially automates coding processes in qualitative content analysis ^29^. The algorithms that drive Leximancer *®* are a commercial secret, but topic modelling software generally uses Latent Dirichelet Allocation algorithms to create statistical models of the distribution and proximity of words in a text or group of texts ^30^. This produces information about associations between them (called ‘concepts’ in Leximancer®) within a natural language corpus ^31^. It identifies empirical regularities and presents these using maps, graphs, and extracted examples. It is important to emphasise that the software undertakes no interpretive activity; it only establishes the relationships between words. Some authors have claimed that these relationship sets, or concepts, are analogous to the categories that ‘manual’ qualitative analysis produces ^32^, even though they require interpretive selection. To undertake analysis using Leximancer® we will follow the procedures set out by Haynes *et al.* ^29^. We will run Leximancer® across the whole data set of included papers ^33^ to identify empirical regularities in natural language data, and ways in which they may be connected ^34^. Searches will be informed by terms from our own coding framework as well as unsupervised Leximancer® coding of a qualitative data set.

### Theoretical interpretation

In both text analytic and manual analysis, we will explore how constructs of the theory have been employed across the qualitative dataset and their contribution to explaining implementation processes. Our approach here will be to perform integrative interpretation of the two bodies of data. We will identify and chart the presence of NPT constructs across the corpus of included papers. We will characterise the ways that these constructs are used to explain core elements of interventions in in practice.

An important problem in directed content analysis is that its results may be restricted by the coding frame in use (i.e., we will only discover that which we are already predisposed to finding). Because an important purpose of this review is to be theoretically informative rather than merely theoretically informed, we will seek insights from the application of existing theory (in this case NPT) to the corpus of data, but also seek to develop and extend these insights. The value of Leximancer® is that it will identify unexpected empirical regularities in natural language data rather than the theory-determined regularities that will be identified in qualitative content analysis.

### Assessment of confidence

AH and CRM will use the GRADE-CERQual (Confidence in the Evidence from Reviews of Qualitative research) approach through the iSoq (Version 1.0) online tool, to assess our confidence in each finding ^35^. GRADE-CERQual assesses confidence in the evidence, based on the following four key components: methodological limitation of included studies, coherence of the review finding, adequacy of the data contributing to a review finding, and relevance of the included studies to the review question. After assessing each of the four components, AH and CRM will make a judgement about the overall confidence in the evidence supporting the review finding. The confidence will be judged as high, moderate, low, or very low, and will be presented in a ‘Summary of Qualitative Findings table’ as per Lewin *et al.* (2018).

### Limitations of our approach

This is an ambitious project, and because of this it brings with it a number of risks and limitations. The first of these is the risk of being overwhelmed with qualitative data. We propose to mitigate this risk by using two kinds of criteria to reduce the number of papers eligible for inclusion in manual analysis: (a) a score of ‘high’ using CASP, and (b) evaluation as theoretically informed or theoretically informative contributions to the literature ^17, 18^. Second, there is the risk that using a published coding framework to support manual analysis will lead to a mechanistic analytic approach. We propose to mitigate this by using additional abductive approaches that actively seek out and identify within the data surprises ^36^, empirical irregularities ^37^, and deviant cases ^38^, that characterise these in relation to NPT, and explain them. A third potential risk is that there are too few theoretically informed or informative papers to give critical mass to a theory-focused review. The experience of our earlier review ^13^ suggests that this is not likely to be a problem. Finally, an important risk is that using Leximancer® to review the results and discussion sections of all papers included in the review will not tell us anything meaningful or useful. Using Leximancer® does not mean that human interpretation is suspended and previous published studies have not suggested that this is an important risk. However, that may be an effect of publication bias. We will therefore treat this as an empirical question, test the data using the software, and critically explore the results. Because this will be done in parallel with the manual analysis, the possible discovery that Leximancer® does not deliver will not pose a threat to the whole project.

### Dissemination and implementation

The primary outcome of this work will be a qualitative evidence synthesis, presented in papers that conform to established reporting standards ^24, 39, 40^. This will lead to peer-reviewed publications and conference and seminar presentations. The workflow and outcomes are described in Figure 2, and will include:

i Overarching qualitative synthesis of key results from papers that that score high against CASP ^24^ in the case of primary studies, or ENTREQ ^40^ in the case of secondary analyses, and are also theoretically informed or informative according to the criteria set out by Kislov *et al.,* ^17, 18^. This will include: (i) controlled and uncontrolled studies of implementation within formal healthcare and social care settings (e.g., primary care/family practice; hospital care); (ii) social care and assisted living; (iii) patient experience studies; and (iv) key results from systematic, scoping, narrative, hermeneutic, meta-ethnography realist and other forms of qualitative evidence synthesis reviews.
ii Overarching topic modelling synthesis of all NPT papers included in the review including those that score in the middle range on CASP and ENTREQ, and those that are categorised as descriptive according to the criteria set out by Kislov *et al.,* ^17, 18^.
iii Theory consolidation paper that links results of the qualitative evidence synthesis to NPT constructs, eliminates constructs that appear redundant, and enhances the theory in practice.

In addition to peer-reviewed journal articles, we will exploit different dissemination pathways: working through two NIHR Applied Research Collaborations in the UK (North Thames, and North-East and North Cumbria), and internationally through the European Implementation Collaborative. Finally, this review will contribute to a programme of work in NIHR North Thames ARC that is intended to lead to the development of a Translational Framework for Implementation Research (the TRIPR study). As findings become available, we will explore their implications with patients and carers, patient and public involvement (PPI) representatives, clinicians, commissioners, and service managers.

## Data availability

No data are associated with this article.
