# Supplementary material for: Translational framework for implementation evaluation and research: Protocol for a qualitative systematic review of studies informed by Normalization Process Theory (NPT)
Source: NIHR Open Res. 2022 Jun 13;2:41. [Version 1] doi: 10.3310/nihropenres.13269.1 (PMC7613237; doi:10.3310/nihropenres.13269.1)
Supplement: Peer review history

# Reviewer report: Reviewer response for version 1

**DOI:** 10.3310/nihropenres.14388.r28669

**Published:** 2022-07-06

**Authors:** Kate Lyle

**Affiliations:** 1 Clinical Ethics, Law and Society (CELS), Wellcome Centre for Human Genetics, University of Oxford, Oxford, UK

This article describes the protocol for a qualitative evidence synthesis of implementation studies that have employed NPT, in order to gain a deeper understanding of how the mechanisms of NPT explain the implementation of healthcare innovations. The evidence provided through the study will enable further theoretical development of NPT, and make an important contribution to implementation science.

 The aims and objectives of the study are clearly described, as are the methods for both identifying and analysing studies. However, the quality assurances processes that will be employed to assess the automated data analysis was less clear, and more detail could be provided to this end.

 In summary, this protocol describes and interesting and important study, that seeks to provide an empirical evidence base to inform theoretical development.

Is the study design appropriate for the research question?

Yes

Is the rationale for, and objectives of, the study clearly described?

Yes

Are sufficient details of the methods provided to allow replication by others?

Yes

Are the datasets clearly presented in a useable and accessible format?

Not applicable

Reviewer Expertise:

Qualitative research. Healthcare implementation.

I confirm that I have read this submission and believe that I have an appropriate level of expertise to confirm that it is of an acceptable scientific standard.

# Reviewer report: Reviewer response for version 1

**DOI:** 10.3310/nihropenres.14388.r28666

**Published:** 2022-06-29

**Authors:** Laura Tarzia

**Affiliations:** 1 Department of General Practice, The University of Melbourne, Carlton, Vic, Australia

This clearly-written article describes the protocol for a systematic review and meta-analysis of qualitative studies informed by Normalization Process Theory (NPT). As the authors assert, NPT is increasingly used as a useful framework for understanding the how, what, where and why of intervention implementation in healthcare settings. It is therefore a timely moment to re-assess how well NPT explains and informs implementation processes. I particularly commend the authors on their willingness to consider potential improvements to NPT and their open-minded approach to learning from the proposed review. 

 My opinion is that this is an excellent, clear and well-written protocol that is likely to result in an interesting and useful systematic review. The methods were described in sufficient detail as to be replicable. The rationale for the review was also well-explained (particularly important given that previous NPT reviews have been undertaken by this team). 

 The use of both "traditional" qualitative techniques and Leximancer is interesting. The authors appear to have carefully considered the advantages and disadvantages of each approach and I am confident the combination of methods will yield valuable results.

 I have some minor suggestions to add clarity to the methods section, as outlined below.

1. Figure 2 says that the COREQ will be used for quality assurance yet this is not described in the text. When will the COREQ be applied? What threshold/score will be selected for inclusion/exclusion? Related to this, it is not clear whether there will be two rounds of quality assurance (one with the COREQ and one with the CASP) to determine inclusion of studies for the manual coding phase, or whether it will just be CASP assessments and the diagram in Fig. 2 needs updating. 
2. I was a little unsure about the definition of "abductive" as simply "searching for unexpected phenomena of interest in the data". This also could be said of inductive analysis. Abductive analysis as I understand it is still a bit contentious in terms of its definition, but I believe it is more about making/testing hypotheses and using the data to either support, critique, or build upon existing theories? Appreciate that the authors may have a particular understanding so am just suggesting that a more detailed description of how they are defining it here would strengthen the manuscript.
3. It would be good to know which research questions are being answered by the manual content analysis and which by Leximancer. Obviously RQ5 specifically refers to the content analysis so that one is clear, but the others don't specify one or the other.
4. In the "Theoretical Interpretation" section the authors state that the content analysis may be limited by the application of NPT as a theoretical framework. This is possible, however, a key part of the abductive approach is that it avoids this particular trap (which I agree is definitely relevant to deductive analysis). This is actually addressed really well in the "Limitations" section, but perhaps would be better placed under "Theoretical Interpretation".

I look forward to reading the publications from this review in future.

Is the study design appropriate for the research question?

Yes

Is the rationale for, and objectives of, the study clearly described?

Yes

Are sufficient details of the methods provided to allow replication by others?

Yes

Are the datasets clearly presented in a useable and accessible format?

Not applicable

Reviewer Expertise:

Qualitative research, qualitative meta-syntheses, healthcare interventions

I confirm that I have read this submission and believe that I have an appropriate level of expertise to confirm that it is of an acceptable scientific standard.
